# An Integrated Photo-Magnetic Sensor Chip Using Giant Magnetoresistance (GMR) and Light-Dependent Resistor (LDR) Technologies Based on Microfabrication Compatibility

**DOI:** 10.3390/mi17050511

**Published:** 2026-04-22

**Authors:** Xuecheng Sun, Xiaolong Chen, Jiao Li, Chunming Ren, Tian Tian, Aiying Guo, Chong Lei

**Affiliations:** 1 School of Mechatronic Engineering and Automation, Shanghai University, Shanghai 200444, China; xl_chen@shu.edu.cn (X.C.); lijiaoshu@shu.edu.cn (J.L.); rcm@i.shu.edu.cn (C.R.); tian25721628@shu.edu.cn (T.T.); 2 School of Microelectronics, Shanghai University, Shanghai 200444, China; 3 National Key Laboratory of Advanced Micro and Nano Manufacture Technology, Department of Micro-Nano Electronics, School of Electronic Information and Electrical Engineering, Shanghai Jiao Tong University, Shanghai 200240, China

**Keywords:** integrated sensor, giant magnetoresistance (GMR), light-dependent resistor (LDR), microfabrication, multifunctional sensor

## Abstract

Single-chip integration technology for multifunctional sensors has become an important development direction due to its low power consumption and versatile functionality. However, the fabrication compatibility between different sensing components remains a key challenge for high-performance integrated sensors, often leading to complex processes and increased costs. This work presents a microfabrication-compatible photo-magnetic integrated sensor chip based on micro–nano processing methods. The integrated sensor chip includes giant magnetoresistance (GMR) and a light-dependent resistor (LDR). The fabrication process was based on standard MEMS fabrication with compatibility and cost-effectiveness. The experimental results demonstrated that the chip can simultaneously realize both optical and magnetic detection with magnetic field sensitivity of 3.74 mV/Oe and photodetection sensitivity of 0.79 μA/(μW/cm^2^) at a 5 V bias. The integrated sensor features high-sensitivity magnetic performance and weak-light detection capability, with promising application in robotics and advanced manufacturing fields.

## 1. Introduction

With the rapid development of robotics, the Internet of Things (IoT), and biomedicine [1,2], the demand for real-time workpiece positioning, surface defect inspection, and health monitoring via optical and magnetic sensing has significantly increased [3]. Traditional single-function sensors have a narrow detection range and poor robustness under multi-variable and high-interference conditions, and thus have inherent limitations. The use of multiple complementary sensors can effectively overcome the limitations of single-type sensors, as they integrate complementary advantages to achieve more comprehensive and reliable detection. Optical and magnetic sensors have been widely used in various fields owing to their versatility. In robotics, magnetic encoders capture the absolute positions of robot joints, and incremental optical encoders deliver high-resolution detection, and their combination further facilitates the miniaturization of robotic devices [4,5]. In IoT low-power sensing nodes such as parking space detectors, optical sensors detect vehicle shadow signals to trigger magnetic sensors, enabling low-power monitoring of parking space occupancy [6]. However, such systems typically rely on multiple discrete sensors connected via data fusion, which often require complex installation and calibration procedures, as well as persistent high power consumption. To address these challenges, multifunctional single-chip integrated sensors have attracted extensive research attention. Realizing diverse sensing functions in a compact form factor by integrating multiple sensing components into a single device has become a major research focus [7,8].

MEMS technology [9,10], CMOS technology [11,12,13,14], and packaging technology [15,16,17] are applied in the fabrication of multifunctional integrated sensors. The integration of multifunctional sensors mainly consists of two modes: System-in-Package (SiP) and System-on-Chip (SoC) [18,19]. In SiP, different sensor dies are laterally assembled on a common substrate with packaging technology, enabling seamless coexistence of heterogeneous materials and a relaxed thermal budget, thereby reducing nonrepetitive engineering costs. However, the trade-off is a large footprint and a reduced upper bandwidth limit caused by parasitic capacitance. On the contrary, SoC sequentially or concurrently manufactures all sensing elements within a single wafer, reducing the interconnection length, decreasing parasitic capacitance, and achieving lower underlying noise and higher bandwidth. However, monolithic integration brings about strict collaborative design constraints: mismatched thermal budgets, protective interlayer deposition, and process flows that require strict optimization.

In the design and fabrication of microsensors, hybrid manufacturing approaches are often utilized to achieve specific structures and functions. CMOS technology enables the cost-effective and highly mature fabrication of semiconductor sensors and on-chip circuits [20]. MEMS processes realize specialized sensors with required deep structures through techniques such as etching [21]. Packaging processes can directly utilize pre-fabricated SoC chips from the above two technologies, enabling the integration of expanded functionalities [22,23].

Roozeboom et al. pioneered SoC and SiP schemes with MEMS technology and packaging technology to integrate magnetic and optical sensors with other functions onto a silicon-based chip with a size of 10 mm × 10 mm [24,25]. They adopted separate sensing layers and sequential fabrication and packaging for different sensor units, leading to a rapid drop in yield as more sensors are integrated. Sutthinet et al. also proposed a diode based on a symmetric silicon p-n junction that can simultaneously detect magnetic field intensity and light intensity, utilizing differential Lorentz force and photoelectric effect [26] with CMOS technology. While CMOS technology greatly simplifies fabrication, it poses challenges in realizing sophisticated structures for high-sensitivity sensors [27,28]. Structures such as cavities and cantilever beams, which are crucial for achieving high sensor sensitivity, still necessitate additional MEMS fabrication processes such as deep reactive ion etching (DRIE), thereby significantly increasing overall process complexity [13,29]. Current integrated photo-magnetic sensors are limited by their structure and fabrication processes, making it difficult to achieve both high performance and satisfactory yield simultaneously.

In recent years, the fabrication of multifunctional environmental sensors based on microfabrication compatibility has become a major research focus [30,31]. Izhar et al. achieved low-cost integration of thermoresistive calorimetric temperature, humidity, and flow sensors on a single chip using a three-step mask lithography process [32]. Simplifying multi-sensor integration through the use of common materials and unified process steps is an effective strategy for enhancing fabrication yield. This process-compatible scheme leverages shared structural features across different sensing elements, allowing multiple sensor structures to be implemented in a single step using standard technologies [32]. To date, most reported works have mainly focused on multilayer planar structures realized by CMOS processes, while the process compatibility of MEMS technologies remains underexplored.

Silicon-based GMR magnetic sensors feature highly mature MEMS fabrication processes and exhibit performance far superior to that of semiconductor magnetic sensors [33,34]. In single-mode magnetic detection applications, researchers typically deposit an opaque metal layer such as Au or Al on the device surface or fabricate a SiO_2_ isolation layer between the GMR and the silicon substrate to isolate the photoelectric effect. However, this also creates favorable conditions for the fabrication of photo-magnetic integrated sensors. Silicon-based photodetectors share identical materials and structures with GMR devices [35]. Dual-physical-quantity detection is achieved by selectively depositing the metal layer on top. The device features two distinct electron transport mechanisms, namely a spin-electron channel and a photon-generated carrier channel. Based on this analysis, GMR sensors and silicon-based photoresistor structures can be fully integrated on a single chip by leveraging their shared materials and structures, eliminating the need for additional preparation of protective or isolation layers [36].

**Figure 1:**
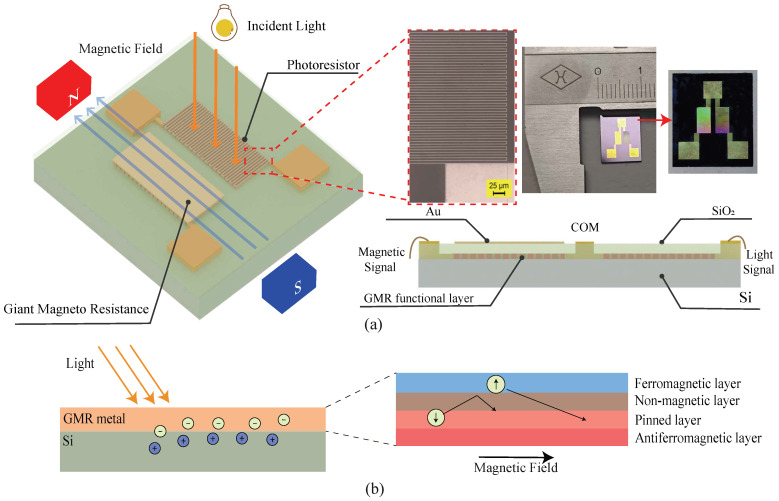
(**a**) Three-dimensional schematic of the integrated device with photoresistor and GMR units, and corresponding device. (**b**) Cross-sectional illustration of photogenerated carrier separation and the four-layer spin-valve structure of the GMR layer.

This study proposes a photo-magnetic detection single-chip integrated sensor by utilizing the giant magnetoresistance effect and a light-dependent resistor (LDR), which incorporate the photosensitive semiconductor principle of the SiO_2_/metal/Si structure based on microfabrication compatibility via a four-mask standard MEMS process. The design and the fabricated sensor chip are presented in Figure 1a. The left panel displays the 3D schematic, illustrating that the chip integrates two functional regions: the GMR section responsible for detecting magnetic fields parallel to the film plane, and the LDR section for sensing incident light perpendicular to the surface. The corresponding microscopic image validates the feasibility of the micro–nano fabrication process. The cross-sectional view further clarifies the four-layer stack structure.

## 2. Design and Fabrication

A schematic of the sensor chip is shown in Figure 1b. When incident light passes through the semi-transparent passivation layer and the GMR line layer, it generates electron–hole pairs in the silicon substrate. The photongenerated electrons can be captured by the GMR metal layer and contribute to the photocurrent. The GMR element adopts a bottom-based spin-valve structure (free layer, nonmagnetic layer, pinned layer, antiferromagnetic layer), in which electron transport follows the two-current model, and electrons are split into spin-up and spin-down channels. When the magnetization of the free layer and pinned layer are parallel, electrons with a matching spin direction pass through the nonmagnetic spacer layer with weak scattering, resulting in a low-resistance state. When the two magnetizations become antiparallel, most electrons experience strong spin-dependent scattering, leading to a high-resistance state.

To separately investigate the individual sensing mechanisms as well as their coupling effects, the chip integrates a GMR magnetic sensor and an LDR light sensor, both fabricated using identical processing steps on a 6-inch GMR wafer via a fully compatible MEMS process (including a GMR metal layer, a Cr/Cu sensing electrode layer, a SiO_2_ protective layer, and a Au lead-out/shielding layer). The only difference is an additional Au light-shielding layer in the magnetic region [37].

**Figure 2:**
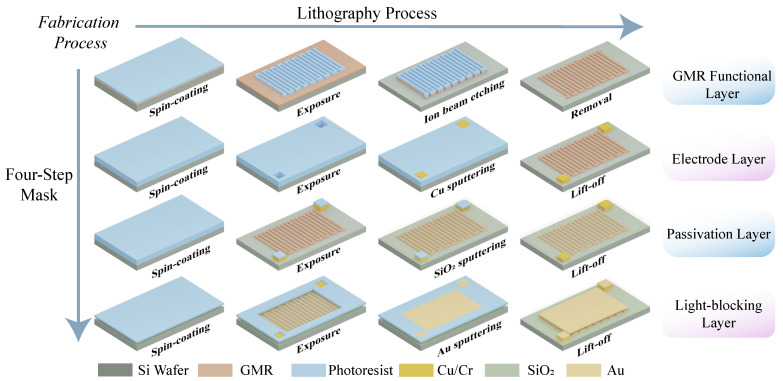
Fabrication process for the integrated photo-magnetic sensor chip.

The fabrication steps for the integrated sensor chip are shown in Figure 2. The device was fabricated on a 6-inch GMR wafer. The first layer is a p-type silicon substrate, which provides mechanical support for the entire sensor structure and acts as a photosensitive layer. Under light exposure, single-crystal silicon substrates effectively absorb photon energy, thereby generating electron–hole pairs, which is a key process for the initiation of photoelectric signals. The second layer is the GMR functional layer, composed of a series of spin-valve stacks, fabricated via sputtering.The spin-valve stack, which is sensitive to weak magnetic fields, consists of a free layer, a nonmagnetic spacer layer, a pinned layer, and an antiferromagnetic layer. It integrates two key features: magnetic sensitivity and semi-transparent conductivity. The third layer consists of metal leads and pad layers for reading photoelectric and magnetoelectric signals. The fourth layer is a passivation layer using SiO_2_ film, which protects the magnetic sensing layer while ensuring light transmission. The optical isolation layer of the GMR sensing unit on the top layer is specially prepared for the GMR sensing unit, and the control of opto-magnetic coupling is achieved by controlling the thickness of the gold film.

Both GMR magnetic sensors and photoresistors adopt a striped structure. For GMR magnetic sensors, the striped structure stabilizes the magnetic domain distribution within the sensor unit and significantly enhances the anisotropic effect, thereby improving the sensor’s responsiveness to magnetic field changes and directly resulting in a higher output signal amplitude. In contrast, for photoresistors, the striped structure first ensures that the photosensitive layer has sufficient light-receiving areas, which is crucial for capturing a sufficient number of photons to be absorbed and excited into photogenerated carriers. Given the process constraints of the employed lithography platform, a 5 μm line dimension ensures no stiction or fracture of the stripes, which has been validated by numerous studies [38,39]. The dimension design is guided by the GMR, which is more challenging to fabricate. Excessively large dimensions reduce the number of chips per wafer, while overly small dimensions increase thermal noise. A resistance of approximately 10 kΩ achieves an optimal balance between these factors. Meanwhile, identical dimensions are adopted for LDR to prevent process-induced failure and facilitate the verification of the coupling relationship in subsequent analysis. The stripe structures of the two devices have consistent design parameters, namely a line width of 5 μm, spacing of 10 μm, a length of 300 μm, and continuous turns of 200, balancing the feasibility of the manufacturing process and overall cost-effectiveness.

The fabrication adopts a four-step mask process: the GMR functional layer (Ta 5 nm /FeNi 2 nm/IrMn 8 nm/CoFe 2 nm/Ru 0.8 nm/CoFe 2 nm/Cu 2.3 nm/CoFe 1.5 nm/FeNi 2 nm/Ta 3 nm) was first sputtered onto the Si substrate and patterned via ion beam etching. The 5 μm metal line structures involve relatively demanding fabrication steps. Continuous dry etching introduces substantial heating, which causes thermal expansion and stress buildup in the photoresist, potentially resulting in cracking. A staged etching process with cooling pauses is therefore utilized to control the temperature and maintain photoresist pattern fidelity. Subsequently, a Cr/Cu bilayer (100 nm) was deposited by magnetron sputtering to form the wiring and electrode layers, with the patterning realized through a lift-off process. Finally, a 300 nm SiO_2_ protective layer and a 150 nm Au light-shielding film were sequentially deposited, and the excess material of both layers was removed via the lift-off process for patterning.Alignment deviations of the gold film may impact the coupling between GMR and LDR structures. The varying photoresponse measured in different GMR devices is likely due to incomplete coverage of the line areas from misalignment, enabling light to pass through the GMR lines and generate photocarriers in the silicon beneath.

## 3. Experimental Details

**Figure 3:**
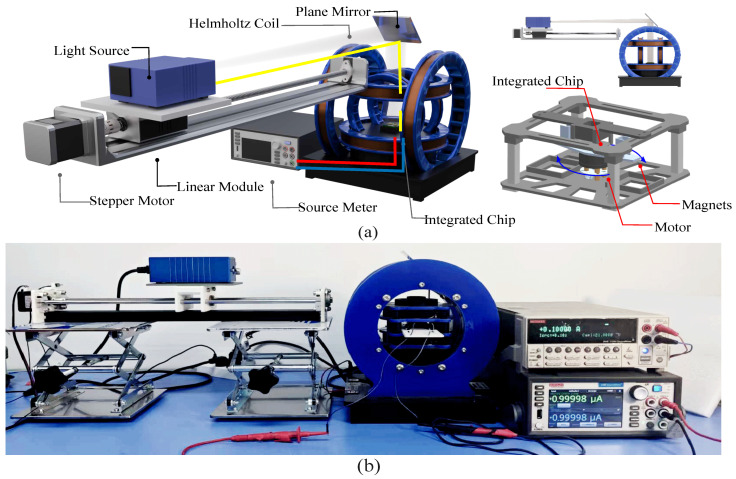
(**a**) Design concept of experimental platform. (**b**) Experimental platform of integrated photo-magnetic sensor chip.

In this work, it is necessary to simultaneously construct the magnetic and optical experimental platforms and characterize their magneto-optical coupling performance. The design of the platform is shown in Figure 3a. The magnetic platform applies an external magnetic field to the GMR chip along the X-axis and Z-axis using Helmholtz coils. The magnitude of the magnetic field is controlled by current. The modulation methods can be expressed as:

(1) $$B={\left(\frac{4}{5}\right)}^{\frac{3}{2}}\frac{\mu_{0}nI}{R}$$

In the formula, $\mu_{0}$ represents the vacuum permeability, *n* indicates the number of coil turns, *I* represents the current magnitude, and *R* represents the coil radius. The magnetic field intensity is calibrated by a gaussmeter. The bias current and output voltage of the chip are uniformly supplied and synchronously collected by a Keithley 2450 digital source meter (Tektronix, Beaverton, OR, USA).

For the optical platform, halogen lamps are selected as the wide-spectrum light source. The position and size of the light spot are adjusted through the reflection of the plane mirror, and the filter group is used to modulate the light wavelength. According to the inverse square law of illuminance,

(2) $$E=\frac{I\cos\theta }{r^{2}}$$

In the formula, *I* represents the light intensity and θ and *r* represent the angle and distance between the light source and the illuminated object, the illuminance is continuously modulated by adjusting the distance between the lamp and the chip through a stepping motor, and the illuminance and optical power of the corresponding wavelength are synchronously calibrated by the color illuminance meter. The bias voltage of the photoresistor and the photocurrent are also read in real time by the Keithley 2450 instrument. The setup of the platform is shown in Figure 3b.

## 4. Results and Discussion

**Figure 4:**
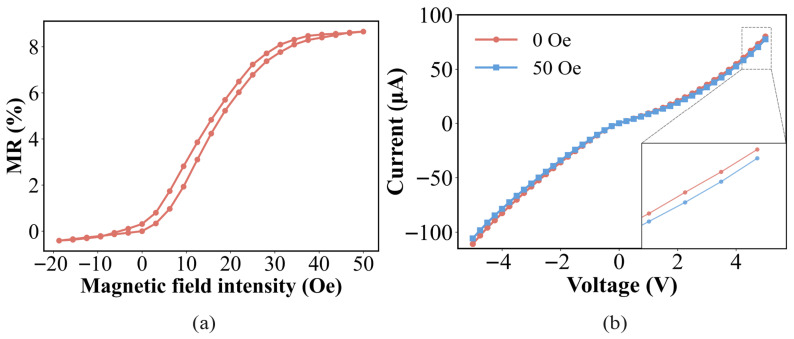
(**a**) Measured MR characteristics of the GMR unit, and (**b**) I–V characteristics of the GMR unit.

Firstly, the magnetoresistance in a dim light environment is excited by a static magnetic field of −20 to 50 Oe to determine the range of the magnetic sensor. Figure 4a shows the MR-H curve of GMR in the absence of light. In the absence of an external magnetic field, the resistance value of GMR is 241.31 kilo-ohms, and the maximum magnetic resistance change rate is 8.68%. Figure 4b shows the volt-ampere characteristics of the GMR device under a magnetic field of 0–50 Oe. It can be seen that as the magnetic field increases, the device has a negative magnetoresistance response, the curve nonlinearity intensifies, and the response under positive and negative voltages is asymmetric. This phenomenon may originate from the Schottky contact formed at the interface between the metal layer and the silicon substrate, which is similar to the origin of the junction effect in the LDR, leading to the introduction of a rectification effect.

**Figure 5:**
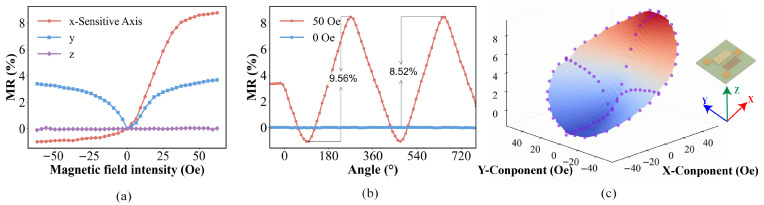
(**a**) MR characteristics of the GMR unit under different directions. (**b**) Angle dependence of MR (%) for the GMR unit. (**c**) Magnetoresistance characteristics of the x–y plane.

In industrial applications, magnetic sensors are often used to detect position and rotation. Figure 5a presents the magnetic field response of the GMR unit in different directions. For the X-axis as the easy axis, the high-sensitivity linear working area ranges from 0 to 40 Oe, and the variation range is from 0 to 8%. After passing the sensitive area, MR gradually increases with the magnetic field intensity. As for the Y-axis, the Y-axis MR will also increase with the magnitude of the magnetic field, and it is not sensitive to the direction of the magnetic field. However, GMR does not respond to the magnetic field in the Z-axis direction.

Figure 5b depicts the variation in the GMR effect with the angle of a rotating 50 Oe magnetic field. The angle θ represents the angle between the magnetic field and the Y-axis. When $\theta =0^{\circ }$ or $\theta =180^{\circ }$, the device is only affected by the magnetic field in the Y-axis direction. When $\theta =90^{\circ }$ or $\theta =270^{\circ }$, the device is only affected by the magnetic field in the X-axis direction. The MR change rates are −1.07% and 8.64% respectively, and the total change rate of the two poles reaches 9.71%, indicating that GMR magnetic coding can effectively detect rotational signals. However, it can also be seen that due to its own characteristics, the sensitivity of magnetic sensors in the pole region is lower than that in the non-pole region. Therefore, the insufficient pole sensitivity of magnetic sensors can be compensated for by optical sensors. Figure 5c shows the angular dependence of GMR with the angle under 50 Oe in the x–y plane.

**Figure 6:**
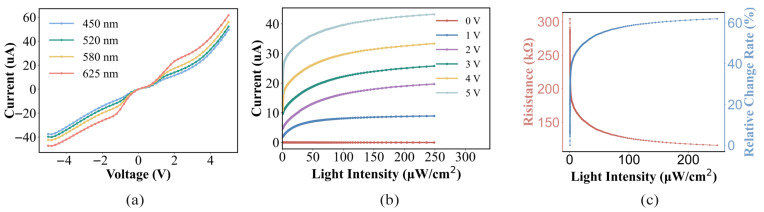
(**a**) Measured I–V characteristics of LDR in dark conditions and when illuminated with different wavelengths of light (108 μW/cm^2^). (**b**) Current–light intensity characteristics of the LDR at a 520 nm wavelength across various voltages. (**c**) Photoresistance and relative resistance change rate characteristics of the device.

In practical applications of LDRs, key considerations include operating conditions, sensitivity to low light, and responsiveness to different light sources. Figure 6a depicts the I-V characteristics of the LDR under different light wavelengths at 108 $\mu W/{\mathrm{cm}}^{2}$. It can be observed that the LDR exhibits a more pronounced response when irradiated with red light at a wavelength of 625 nm. LDRs exhibit both a photoconductive effect and a photovoltaic effect: at high bias voltages, the photoconductive effect dominates—with increasing light intensity, the number of carriers increases, leading to a decrease in resistance. At low bias voltages, however, the photovoltaic effect prevails, accompanied by a junction effect and the presence of a dark current. Figure 6b illustrates the relationship between photocurrent and optical power density of a photodetector under different bias voltages. For the same optical power, the higher the voltage, the larger the current. This is because a forward bias reduces the barrier height of the junction, accelerates the diffusion speed of carriers, and improves the carrier collection efficiency, thereby increasing the current. Meanwhile, the response also increases with the increase in voltage, indicating that the detector is more sensitive to light at higher voltages. From the curve characteristics, the higher the voltage, the steeper the curve slope, which means that the photocurrent grows faster with the optical power at higher voltages. However, at high optical power and high voltage, the curve slope becomes gentler. This may be due to the carriers reaching the material limit or the influence of electrode contact resistance, but no complete saturation is observed.

To explore the sensitivity of the LDR in low-light conditions, Figure 6c shows the characteristics of the photoresistance and relative resistance change rate of the LDR under a working voltage of 5 V. In particular, in a weak-light environment with a light intensity of 10 μW/cm^2^, the LDR exhibits a response of 3.02 mA/W and a resistance change rate of 56%. The dark resistance is about 300 kΩ.

**Figure 7:**
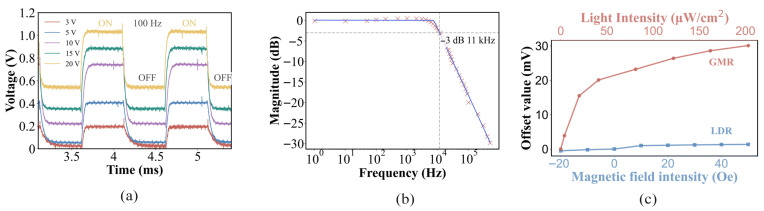
(**a**) Time response of the LDR at various applied voltages. (**b**) Frequency response of the LDR at 10V. (**c**) The crosstalk between the LDR and GMR units.

The time response characteristic of an LDR determines its performance in high-speed detection scenarios. Figure 7a presents the transient response curves of the LDR under different bias voltages, with the test conducted at a frequency of 100 Hz. The experimental data show that when switching from the dark state to the light state, the response time is 11 μs under a 3 V bias, which shortens to 4.9 μs under a 10 V bias. When transitioning from the light state to the dark state, the recovery time remains stable at 10 μs which is governed by the recombination dynamics of photogenerated carriers and carrier extraction at the Schottky interface. After light removal, photocarriers recombine rapidly, and the built-in electric field at the metal–silicon junction further extracts residual carriers. Due to the inherent structural characteristics of the device, the recovery time limits the overall response performance of the LDR. Under low-light illumination, the response current exhibits a variation rate of less than 3.5% over a 10-minute continuous operation, demonstrating excellent long-term stability. Figure 7b presents the frequency response of the LDR at a 10 V bias, with a measured 3 dB bandwidth of 11.3 kHz. Compared with nanosecond-scale silicon-based photodiodes, the metal layer forms only single-interface contact with the silicon layer, which restricts the carrier collection rate. To further improve the response speed, structural optimization of the LDR remains a feasible approach.

To investigate how Au mitigates the light-induced decoupling of the magnetic response, the test platform is employed to apply light and magnetic signals simultaneously. Figure 7c illustrates the offset voltage response of the GMR and LDR units under combined stimuli of magnetic field intensity and light intensity. Since the Au film was not sufficiently thick to fully block incident light, the overall resistance of the unit decreases with increasing light intensity. In contrast, the LDR unit exhibited no response to changes in magnetic field intensity.

## 5. Conclusions

The chip-level integrated photo-magnetic sensor with microfabrication compatibility outperforms existing integration schemes in terms of detection performance, as shown in Table 1. The experimental results demonstrated that the integrated chip achieved a light detection sensitivity of 0.79 μA/(μW/cm^2^) and a magnetic field sensitivity of 3.74 mV/Oe.

In summary, this work provides a feasible technical route for high-performance photo-magnetic integrated sensors compatible with standard microfabrication processes that address the practical application demands for multifunctional sensors.

## Figures and Tables

**Table 1:** Comparison of key performance metrics for integrated photo-magnetic sensors.

Sensor	Size	Parameter	Principle	Sensitivity (Sensor Only)
[24]	10 mm × 10 mm	Magnetic	Hall effect	0.038 μV/Oe
		Optical	Photoresistor	0.6 μV/Lux
			Photodiode	2.3 nA/Lux (0 V)
[26]	430 μm × 200 μm	Magnetic	Lorentz force	0.0128 μA/Oe
		Optical	p-n junction diode	0.576 μA/(μW/cm^2^)
This work	1.5 mm × 3 mm	Magnetic	Giant magnetoresistance	3.74 mV/Oe
		Optical	Photoresistor	0.79 μA/(μW/cm^2^)

## Data Availability

The original contributions presented in this study are included in the article. Further inquiries can be directed to the corresponding authors.

## References

[1] Cheshire C., Gliese F., Bertele F., Ammann U. (2022). Overview of a Multifunctional Sensor Module for Electric Drives Based on Contactless Measurement Techniques. Proceedings of the 2022 International Conference on Electrical Machines (ICEM), Valencia, Spain, 5–8 September 2022.

[2] Deb Majumder B., Roy J.K., Padhee S. (2019). Recent Advances in Multifunctional Sensing Technology on a Perspective of Multi-Sensor System: A Review. IEEE Sensors J..

[3] Khan B., Khan W., Masrur M.H., Khalid R.T., Awais M., Khan B., Khoo B.L., Abdullah S. (2025). Hybrid Sensor Integration in Wearable Devices for Improved Cardiovascular Health Monitoring. J. Sci. Adv. Mater. Devices.

[4] Tobita K., Ohira T., Kajitani M., Kanamori C., Shimojo M., Ming A. (2005). A Rotary Encoder Based on Magneto-Optical Storage. IEEE/ASME Trans. Mechatron..

[5] Kim S.K., Kim H.J., Lee S., Park S.H., Lee K.C. (2016). Detection of Absolute Position for Magneto-Optical Encoder Using Linear Table Compensation. J. Korean Soc. Precis. Eng..

[6] Sifuentes E., Casas O., Pallas-Areny R. (2011). Wireless Magnetic Sensor Node for Vehicle Detection with Optical Wake-Up. IEEE Sensors J..

[7] Zhao W., Alcheikh N., Mbarek S.B., Younis M.I. (2022). Multi-Functional Resonant Micro-Sensor for Simultaneous Magnetic, CO_2_, and CH_4_ Detection. J. Appl. Phys..

[8] Zhang J., Cheng C., Zhang H., Zhou J., Liu H., Zhang H., Chen L., Zhao T. (2024). Dual-Parameter and High-Density Sensor Array Based on a-IGZO Thin Film Transistors. IEEE Electron Device Lett..

[9] Rusli N.I., Vincentini I.P., Ceyssens F., Kraft M. (2021). Multi-Sensor Chip for Monitoring Key Parameters in Bioprocesses. IEEE Sensors J..

[10] Cheng C., Yao J., Xue H., Lu Y., Wang J., Chen D., Chen J. (2022). A MEMS Resonant Differential Pressure Sensor with High Accuracy by Integrated Temperature Sensor and Static Pressure Sensor. IEEE Electron Device Lett..

[11] Chang C.C., Hong P.H., Yeh S.K., Lin Y.C., Lai M.F., Fang W. (2020). Environmental Sensing Hub on Single Chip Using Double-Side Post-CMOS Processes. Proceedings of the 2020 IEEE 33rd International Conference on Micro Electro Mechanical Systems (MEMS), Vancouver, BC, Canada, 18–22 January 2020.

[12] Chien T.L., Huang Y., Shih F., Fang W. (2023). Monolithically and Vertically Integrated Environmental Sensing Hub with Novel Air-Based Humidity Sensor Design. Proceedings of the 2023 IEEE 36th International Conference on Micro Electro Mechanical Systems (MEMS), Munich, Germany, 15–19 January 2023.

[13] Mansoor M., Haneef I., Akhtar S., Rafiq M., De Luca A., Ali S., Udrea F. (2016). An SOI CMOS-Based Multi-Sensor MEMS Chip for Fluidic Applications. Sensors.

[14] Lakshminarayana S., Park H., Jung S. (2025). Emerging Technologies in CMOS Integrated Sensing System-On-Chip: A Review. IEEE Sensors Rev..

[15] Jiang C., Chen W., Yin J., Chen X., Zhang X., Wu Y., Lou L., Xie J., Tang B., Jin Q. (2024). Batch Fabrication and Testing of Multiparameter Integrated Sensor for Marine Environmental Measurements. IEEE Sensors J..

[16] Kock A., Wimmer-Teubenbacher R., Sosada-Ludwikovska F., Rohracher K., Wachmann E., Herold M., Welden T.V., Min Kim J., Ali Z., Poenninger A. (2019). 3D-Integrated Multi-Sensor Demonstrator System for Environmental Monitoring. Proceedings of the 2019 20th International Conference on Solid-State Sensors, Actuators and Microsystems & Eurosensors XXXIII (TRANSDUCERS & EUROSENSORS XXXIII), Berlin, Germany, 23–27 June 2019.

[17] Zhang S., Li Z., Zhou H., Li R., Wang S., Paik K.W., He P. (2022). Challenges and Recent Prospectives of 3D Heterogeneous Integration. E-Prime Electr. Eng. Electron. Energy.

[18] Koh W. (2005). System in Package (SiP) Technology Applications. Proceedings of the 2005 6th International Conference on Electronic Packaging Technology, Shenzhen, China, 30 August–2 September 2005.

[19] Tummala R., Madisetti V. (1999). System on Chip or System on Package?. IEEE Des. Test Comput..

[20] De Luca A., Haneef I., Coull J.D., Ali S.Z., Falco C., Udrea F. (2015). High-Sensitivity Single Thermopile SOI CMOS MEMS Thermal Wall Shear Stress Sensor. IEEE Sensors J..

[21] Dong J., Long Z.J., Jiang H., Sun L. (2017). Monolithic-Integrated Piezoresistive MEMS Accelerometer Pressure Sensor with Glass-Silicon-Glass Sandwich Structure. Microsyst. Technol..

[22] Lee K.W., Noriki A., Kiyoyama K., Fukushima T., Tanaka T., Koyanagi M. (2011). Three-Dimensional Hybrid Integration Technology of CMOS, MEMS, and Photonics Circuits for Optoelectronic Heterogeneous Integrated Systems. IEEE Trans. Electron Devices.

[23] Wang J., Li X. (2013). Package-Friendly Piezoresistive Pressure Sensors with on-Chip Integrated Packaging-Stress-Suppressed Suspension (PS^3^) Technology. J. Micromechanics Microengineering.

[24] Roozeboom C.L., Hopcroft M.A., Smith W.S., Sim J.Y., Wickeraad D.A., Hartwell P.G., Pruitt B.L. (2013). Integrated Multifunctional Environmental Sensors. J. Microelectromechanical Syst..

[25] Roozeboom C.L., Hill B.E., Hong V.A., Ahn C.H., Ng E.J., Yang Y., Kenny T.W., Hopcroft M.A., Pruitt B.L. (2015). Multifunctional Integrated Sensors for Multiparameter Monitoring Applications. J. Microelectromechanical Syst..

[26] Sutthinet C., Phetchakul T., Luanatikomkul W., Poyai A. (2015). Multi-Sensor Diode for Magnetic Field and Photo Detection. Proceedings of the 10th IEEE International Conference on Nano/Micro Engineered and Molecular Systems, Xi’an, China, 7–11 April 2015.

[27] Chien T.L., Lee Y.C., Chou T., Lin Y.Y., Chen H.Y., Fang W. (2022). Fabrication and Integration of Soc Environment Sensing Hub with Gas/Pressure/Temperature Sensors. Proceedings of the 2022 IEEE 35th International Conference on Micro Electro Mechanical Systems Conference (MEMS), Tokyo, Japan, 9–13 January 2022.

[28] Lin Y.C., Hong P.H., Yeh S.K., Chang C.C., Fang W. (2020). Monolithic Integration of Pressure/Humidity/Temperature Sensors for CMOS-Mems Environmental Sensing Hub with Structure Designs for Performances Enhancement. Proceedings of the 2020 IEEE 33rd International Conference on Micro Electro Mechanical Systems (MEMS), Vancouver, BC, Canada, 18–22 January 2020.

[29] Mansoor M., Haneef I., Akhtar S., Rafiq M.A., Ali S.Z., Udrea F. (2014). SOI CMOS Multi-Sensors MEMS Chip for Aerospace Applications. Proceedings of the IEEE SENSORS 2014 Proceedings, Valencia, Spain, 2–5 November 2014.

[30] Izhar, Xu W., Tavakkoli H., Zhao X., Lee Y.K. (2022). CMOS Compatible MEMS Multienvironmental Sensor Chip for Human Thermal Comfort Measurement in Smart Buildings. IEEE Trans. Electron Devices.

[31] Xu W., Hong L., Pan X., Izhar (2024). Monolithically Integrated Bidirectional Flow Sensor and Stacked Temperature/Humidity Sensor Based on CMOS-Compatible MEMS Technology. IEEE Trans. Instrum. Meas..

[32] Izhar, Xu W., Tavakkoli H., Cabot J., Zhao X., Duan M., Lee Y.K. (2021). Single-Chip Integration of CMOS Compatible Mems Temperature/Humidity and Highly Sensitive Flow Sensors for Human Thermal Comfort Sensing Application. Proceedings of the 2021 21st International Conference on Solid-State Sensors, Actuators and Microsystems (Transducers), Orlando, FL, USA, 20–24 June 2021.

[33] Jeng J.T., Trinh X.T., Hung C.H., Lu C.C. (2019). Quasi-Static Current Measurement with Field-Modulated Spin-Valve GMR Sensors. Sensors.

[34] Ardiyanti H., Mabarroh N., Wibowo N.A., Istiqomah N.I., Tumbelaka R.M., Ulil Absor M.A., Suharyadi E. (2023). New Design of a Commercial Chip-Based GMR Sensor with Magnetite Nanoparticles for Biosensing Applications. J. Sci. Adv. Mater. Devices.

[35] Ho J., Wong K. (1996). Bandwidth enhancement in silicon metal-semiconductor-metal photodetector by trench formation. IEEE Photonics Technol. Lett..

[36] Zu B., Lu B., Guo Y., Xu T., Dou X. (2014). Simple Metal/SiO_2_/Si Planar Photodetector Utilizing Leakage Current Flows through a SiO_2_ Layer. J. Mater. Chem. C Mater. Opt. Electron. Devices.

[37] Tan X., Li Z., Gao X., Liu W., Zhao M., Ren Y., Ding Q., Li B., Song Y., Zheng B. (2025). Enhancing Magnetic Bead Detection: Structural Innovations in GMR Sensors. IEEE Sensors J..

[38] Wang Z., Sun X., Natalia A., Tang C.S.L., Ang C.B.T., Ong C.A.J., Teo M.C.C., So J.B.Y., Shao H. (2020). Dual-Selective Magnetic Analysis of Extracellular Vesicle Glycans. Matter.

[39] Sun X., Lei C., Guo L., Zhou Y. (2016). Separable detecting of *Escherichia coli* O157H:H7 by a giant magneto-resistance-based bio-sensing system. Sens. Actuators B Chem..

